# Food Insecurity, Hunger, and Obesity Among Informal Caregivers

**DOI:** 10.5888/pcd12.150129

**Published:** 2015-10-08

**Authors:** Willi Horner-Johnson, Konrad Dobbertin, Sheetal Kulkarni-Rajasekhara, Erin Beilstein-Wedel, Elena M. Andresen

**Affiliations:** Author Affiliations: Konrad Dobbertin, Sheetal Kulkarni-Rajasekhara, Erin Beilstein-Wedel, Elena M. Andresen, Institute on Development and Disability, Oregon Health and Science University, Portland, Oregon.

## Abstract

**Introduction:**

Increasing numbers of US residents rely on informal caregiving from friends and family members. Caregiving can have substantial health and financial impacts on caregivers. This study addressed whether those impacts include adverse nutritional states. Specifically, we examined household food insecurity, individual hunger, and obesity among caregivers compared with noncaregivers.

**Methods:**

We analyzed 2012 Behavioral Risk Factor Surveillance System data from Oregon. The Caregiving Module was administered to a random subset of 2,872 respondents. Module respondents included 2,278 noncaregivers and 594 caregivers providing care or assistance to a friend or family member with a health problem or disability. We used multivariable logistic regression to assess associations between caregiving status and each of our dependent variables.

**Results:**

Caregivers had significantly greater odds of reporting household food insecurity (odds ratio [OR] = 2.10, *P* = .003) and personal hunger (OR = 2.89, *P* = .002), even after controlling for income and other correlates of food insecurity. There were no significant differences in obesity between caregivers and noncaregivers.

**Conclusion:**

Caregiving is associated with increased risk of food insecurity and hunger in Oregon, suggesting that careful attention to the nutritional profile of households with family caregivers is needed in this population.

## Introduction

As the United States population ages, the number of older people requiring long-term services and supports increases (1). Much of that care is provided informally by family members and friends (2). Caregiving is associated with substantial physical and mental health effects on caregivers (1,3–9). Providing unpaid care can also create a financial burden for caregivers (2,9).

Financial strain in caregiver households may lead to food insecurity and hunger. Food insecurity is defined as “limited or uncertain availability of nutritionally adequate and safe foods” (10). Hunger is “the uneasy or painful sensation caused by lack of food” or “recurrent and involuntary lack of access to food” (10). In 2006, the US Department of Agriculture adopted the term “very low food security” instead of hunger to describe households whose members have reduced food intake because they cannot afford to eat more (11). However, some policy groups have continued to use the term hunger because it is more understandable by the general public (10), and some data sources assess both household food insecurity and personal hunger.

Income, education, race/ethnicity, functional limitations, and presence of working-age adults with disabilities, adults with chronic physical or mental health conditions, or children in the household are predictors of food insecurity (12–17). Some research in other countries has found food insecurity among caregivers due to reduced income and increased expenses associated with caregiving (18). However, the relationship between caregiving and food insecurity has received little attention in the United States. Financial and other stresses of caregiving may also contribute to obesity via consumption of less varied and poorer quality foods and limited time for physical activity (19–21

This study sought to examine the relationship between caregiver status, household food insecurity, individual hunger, and obesity in Oregon State. We hypothesized that caregivers would be more likely than noncaregivers to experience these 3 conditions.

## Methods

We analyzed 2012 Behavioral Risk Factor Surveillance System (BRFSS) data from Oregon. The BRFSS is a representative telephone-based survey of health behaviors and health risks that is conducted by the Centers for Disease Control and Prevention (CDC) in partnership with health departments in all 50 US states, the District of Columbia, and US territories. In 2005, a Caregiving Module was created to describe caregiver and care recipient characteristics (22,23). The Caregiving Module was implemented for the first time in Oregon in 2012. We analyzed responses from Oregon survey participants who received this module.

The Caregiving Module was administered to a random subset of 2,872 Oregon BRFSS participants, including respondents with both landline and cellular telephones. Module respondents included 594 caregivers and 2,278 noncaregivers. We excluded 119 individuals with missing data for the core independent variables described below, yielding a sample of n = 2,753. For regression analyses we further excluded individuals with missing data for the 3 outcomes in question, resulting in 3 separate analytic samples for food insecurity (total n = 2,054; caregivers n = 450; noncaregivers n = 1,604); hunger (total n = 2,051; caregivers n = 450; noncaregivers n = 1,601); and obesity (total n = 2,625; caregivers n = 549; noncaregivers n = 2,076).

### Measures

We used 3 dependent variables: whether or not a respondent experienced food insecurity in their household during the past 12 months (yes or no), a respondent personally experienced hunger during the past 12 months (yes or no), and a respondent was obese (yes or no). The presence of household food insecurity was identified by an affirmative answer to any of 3 questions about not having enough food, not being able to afford balanced meals, or skipping meals and cutting meal size due to a lack of food or insufficient money to buy food. Hunger was identified by a positive response to at least one of 2 questions that asked respondents if they personally had done the following things because of a lack of money: eaten less than they felt they should, or were hungry and did not eat. Obesity was identified by an adult body mass index of 30 or higher, based on self-reported height and weight (kg/m^2^).

Our primary independent variable was caregiver status (yes or no); noncaregivers served as the reference category. Caregivers were identified with the first question in the Caregiving Module that asks about providing care or assistance in the past month to a friend or family member who has a health problem or disability. Core covariates included respondent age, sex, race/ethnicity, self-reported health status, and annual household income. We also tested respondent disability status, education, employment status, number of adults in the household, and presence of children in the household for possible inclusion in our models.

We analyzed age as a continuous variable. Sex was coded in 2 groups (men vs women); men were the reference group. Our analytic sample was largely non-Hispanic white, similar to the overall demographics of the state; therefore, we dichotomized race/ethnicity into non-Hispanic white (reference) versus all other racial/ethnic groups. We also dichotomized self-reported health status categories into excellent, very good, or good (reference) versus fair or poor. Family income is a correlate of food insecurity (16,17,20). However, the income variable had more missing data than was the case for other covariates. Thus, we included a “missing” category for the income covariate. Income categories were $50,000 or more per year (reference); $35,000 to less than $50,000; $25,000 to less than $35,000; $15,000 to less than $25,000; less than $15,000; and missing.

In addition to caring for someone else with a disability, caregivers may have disabilities themselves (2). We therefore examined disability status as a potential covariate. Presence of a disability was identified by an affirmative response to one or both of the following standard BRFSS questions (24): 1) Are you limited in any way in any activities because of physical, mental, or emotional problems? 2) Do you now have any health problem that requires you to use special equipment such as a cane, wheelchair, a special bed, or a special telephone? Education consisted of 4 groups: 1) did not graduate high school, 2) graduated high school, 3) attended college or technical school, 4) graduated from college or technical school. We dichotomized employment status into those currently employed versus individuals who were unemployed or out of the workforce (eg, retired). Number of adults in the household was analyzed in 4 categories: 1 adult, 2 adults, 3 adults, and 4 or more adults. We analyzed children in the household as a dichotomous variable: any children (under age 18) versus none.

Although not included in our regression models comparing caregivers with noncaregivers, we did conduct descriptive analyses of care recipient characteristics, caregiving intensity and duration, and impacts of caregiving as background for understanding caregiver situations. Variables assessed in the Caregiver Module included the care recipient’s age, sex, relationship to caregiver, health problem, and area of greatest need for assistance. Additional questions ascertained number of hours per week spent caregiving, number of months as a caregiver, and greatest difficulty caused by caregiving.

### Analyses

We performed both bivariate and multivariate survey weighted logistic regression to determine the association between caregiver status and household food insecurity, respondent hunger, and respondent obesity. All potential independent variables were entered simultaneously in the multivariate model. Independent variables were retained in the final model if they were significant, with the exception of key variables we had decided a priori would be retained regardless of significance. The latter category included caregiver status and the basic demographic variables: age, sex, and race/ethnicity. A *P* value of less than .05 was selected as the cutoff for significance. We used Stata version 12.1 (StataCorp, LP) to account for the complex survey design of the BRFSS. Additionally, because odds ratios [ORs] may be inflated for more common events, we applied a corrective factor to our adjusted ORs for associations of caregiving with food insecurity, hunger, and obesity to more closely approximate risk ratios (25).

## Results

After excluding observations with missing data for age, sex, race/ethnicity, and health status, 19.4% of our sample identified themselves as caregivers. Compared with noncaregivers, higher proportions of caregivers were female and reported fair or poor health. Caregivers also had a greater tendency to have lower income (Table 1). Additionally, larger proportions of caregivers reported food insecurity, hunger, and obesity (24.4%, 11.5%, and 30.9%, respectively) relative to noncaregivers (14.9%, 4.6%, and 25.4%, respectively). All respondents classified as food insecure responded affirmatively to at least 2 of the questions. Respondents who reported hunger had affirmative responses to all 3 of the household food insecurity questions in addition to at least one of the hunger questions.

**Table 1:** Characteristics of Caregivers and Noncaregivers, 2012 Oregon Behavioral Risk Factor Surveillance System

Characteristic	n (Weighted %)		
	Caregivers	Noncaregivers	Total
**Age (mean, standard error)**	50.8 (1.03)	47.2 (0.57)	47.9 (0.50)
**Sex**			
Male	197 (40.5)	923 (50.2)	1,120 (48.3)
Female	379 (59.5)	1,254 (49.8)	1,633 (51.7)
**Race/ethnicity**			
White, non-Hispanic	496 (87.8)	1,876 (84.8)	2,372 (85.4)
Other	80 (12.2)	301 (15.2)	381 (14.6)
**Health status**			
Excellent, very good, or good	451 (76.4)	1,797 (84.7)	2,248 (83.1)
Fair or poor	125 (23.6)	380 (15.3)	505 (16.9)
**Family income, $**			
Less than 15,000	64 (13.8)	180 (9.5)	244 (10.3)
15,000 to <25,000	85 (16.7)	337 (15.6)	422 (15.8)
25,000 to <35,000	56 (10.7)	195 (8.5)	251 (8.9)
35,000 to <50,000	81 (11.9)	302 (13.2)	383 (13.0)
50,000 or more	220 (33.3)	869 (37.4)	1,089 (36.6)
Missing	70 (13.6)	294 (15.8)	364 (15.4)
Total	576 (19.4)	2,177 (80.6)	2,753 (100)

Nearly two-thirds of care recipients were aged 61 years or older, 59.8% were women, and approximately 80% were a relative of the caregiver (Table 2). Many different health problems necessitated care; Alzheimer’s disease, arthritis, cancer, and heart disease were the leading causes. Stress was the most common difficulty caregivers attributed to their caregiving, but 37.5% said caregiving resulted in no additional difficulties.

**Table 2:** Care Recipient and Caregiving Characteristics (n = 576), 2012 Oregon Behavioral Risk Factor Surveillance System

Characteristic	n (Weighted %)
**Care recipient age, y**	
≤20	19 (4.1)
21–30	21 (4.6)
31–40	23 (5.9)
41–50	33 (8.3)
51–60	65 (14.3)
61–70	83 (14.7)
71–80	99 (15.7)
≥81	233 (32.4)
Total	576 (100)
**Care recipient sex**	
Male	236 (40.2)
Female	337 (59.8)
Total	573 (100)
**Care recipient relationship to caregiver**	
Parent/parent-in-law	206 (38.7)
Child/grandchild	52 (7.2)
Spouse	116 (17.4)
Other relative	74 (14.9)
Nonrelative	124 (21.8)
Total	572 (100)
**Care recipient health problem or illness**	
Arthritis	26 (5.8)
Cancer	29 (6.5)
Heart disease	39 (8.8)
Alzheimer’s disease	51 (7.8)
Other^a^	391 (71.1)
Total	536 (100)
**Care recipient needs most help with**	
Taking care of self^b^	76 (14.3)
Taking care of personal living spaces^c^	163 (34.6)
Transportation outside of the home	133 (21.5)
Other	167 (29.6)
Total	539 (100)
**Greatest difficulty faced by caregivers**	
Creates a financial burden	40 (6.9)
Not enough time for yourself	40 (8.3)
Not enough time for your family	24 (5.4)
Creates stress	135 (24.7)
Affects family relationships	35 (5.3)
Other	71 (11.8)
No difficulty	213 (37.5)
Total	558 (100)
**Hours per week of caregiving**	
≤2	103 (17.1)
3–5	110 (23.6)
6–10	102 (19.6)
11–39	109 (24.0)
≥40	78 (15.7)
Total	502 (100)
**Months of caregiving**	
≤3	112 (22.8)
4–12	102 (22.0)
13–24	88 (15.6)
25–60	119 (21.4)
≥60	119 (18.2)
Total	540 (100)

a All other health problems and illnesses were less than 5% of the sample.

b Taking care of self refers to, for example, eating, dressing, or bathing.

c Taking care of personal living spaces refers to cleaning, managing money, or preparing meals.

In bivariate (crude) regression models, caregivers had nearly twice the odds of noncaregivers of being in food insecure households (OR = 1.85; 95% confidence interval [CI], 1.21–2.80). Caregivers also had nearly 3 times the odds of noncaregivers of personally experiencing hunger (OR = 2.70; 95% CI, 1.41–5.20). Caregivers did not have significantly increased odds of being obese (OR = 1.31; 95% CI, 0.97–1.77). In multivariate (adjusted) models, these associations remained largely unchanged (Table 3). Disability status (of survey respondent), education, employment, number of adults in household, and presence of children in household were not significantly associated with our dependent variables in the context of other variables in the models, nor did they affect the significance of the association between caregiving and our dependent variables. In the interest of parsimony we excluded these variables from the results (Table 3). Application of the corrective factor (25) for caregiver effects reduced the final adjusted odds ratios somewhat but did alter overall patterns of food insecurity (OR = 1.80; 95% CI, 1.24–2.51), hunger (OR = 2.66; 95% CI, 1.44–4.67), and obesity (OR = 0.15; 95% CI, 0.92–1.42).

**Table 3:** Adjusted^a^ Odds of Household Food Insecurity, Personal Hunger, and Obesity^b^ for Caregivers, 2012 Oregon Behavioral Risk Factor Surveillance System

Variables	Food Insecurity (n = 1,792)		Hunger (n = 1,789)		Obesity (n = 2,309)	
	AOR (95% CI)	*P *Value	AOR (95% CI)	*P *Value	AOR (95% CI)	*P *Value
**Caregiver**						
No	1 [Reference]					
Yes	2.10 (1.29–3.42)	.003	2.89 (1.47–5.68)	.002	1.21 (0.89–1.66)	.23
**Age (y)**	0.95 (0.94–0.97)	<.001	0.95 (0.93–0.97)	<.001	1.01 (1.00–1.02)	.01
**Sex**						
Male	1 [Reference]					
Female	1.14 (0.71–1.84)	.60	1.41 (0.64–3.09)	.40	0.98 (0.76–1.27)	.90
**Race/ethnicity**						
Non-Hispanic white	1 [Reference]					
Other	1.61 (0.76–3.41)	.22	1.66 (0.53–5.21)	.38	1.41 (0.88–2.25)	.15
**Health status**						
Excellent, very good, or good	1 [Reference]					
Fair/poor	2.66 (1.69–4.19)	<.001	2.42 (1.33–4.41)	.004	2.15 (1.56–2.96)	<.001
**Family income, $**						
≥50,000	1 [Reference]					
35,000 to <50,000	4.50 (1.66–12.19)	.003	1.58 (0.28–9.02)	.61	0.98 (0.65–1.46)	.90
25,000 to <35,000	17.09 (6.12–47.69)	<.001	30.29 (6.92–132.65)	<.001	0.79 (0.48–1.28)	.34
15,000 to <25,000	17.59 (7.07–43.74)	<.001	14.29 (3.62–56.42)	<.001	0.82 (0.56–1.19)	.29
<15,000	30.68 (11.80–79.77)	<.001	45.58 (11.95–173.86)	<.001	0.99 (0.62–1.57)	.96
Missing	9.43 (3.43–25.94)	<.001	3.85 (0.74–19.99)	.11	0.87 (0.56–1.34)	.52

Abbreviations: AOR, adjusted odds ratio; CI, confidence interval.

a Adjusted for other variables shown in table.

b Defined as body mass index of 30 kg/m^2^ or more.

Some of the covariates in the final adjusted models did demonstrate significant associations. Age was negatively associated with food insecurity and hunger (risk decreased with age) but positively associated with obesity (risk increased with age). Those who reported fair or poor health were significantly more likely to experience all 3 outcomes. Income was strongly associated with both food insecurity and hunger, but not obesity (Table 3).

## Discussion

As hypothesized, caregivers were significantly more likely than noncaregivers to experience household-level food insecurity, individual-level hunger, or both. That finding remained true when controlling for other factors that were also significantly associated with food insecurity and hunger (age, race/ethnicity, health status, and income). Caregivers were also somewhat more likely to be obese, but that difference was not significant. Although income was strongly associated with both food insecurity and hunger, controlling for differences in family income did not account for the significant relationships between caregiving and food insecurity and hunger. Prior research in the general US population has also found that food insecurity is not fully explained by differences in family income (20). We speculate that caregivers in particular may encounter additional expenses that make an otherwise adequate family income no longer sufficient to afford enough food. Although previous studies have found indications of food insecurity among caregivers in lower and middle income countries (18), our results indicate that food insecurity is also a serious issue for caregivers in a wealthy country.

Food insecurity, including hunger (very low food security), is associated with a range of poor adult health outcomes, including inflammation, chronic disease, and poorer control of chronic conditions such as diabetes (26–28). Thus, the high prevalence of food insecurity among caregivers is cause for concern. Caregiving itself has been associated with poor physical and mental health (6,9,19). Food insecurity among caregivers may contribute to or exacerbate those health problems. Healthy People 2020 (29) includes objectives on promoting health and well-being of caregivers and reducing unmet needs for caregiver support services among unpaid caregivers. Based on our findings, we recommend that attention to food insecurity be considered as a component in addressing each of these objectives.

Our analysis did not find an association between caregiver status and obesity. However, prior research has found relationships between food insecurity and obesity, particularly among women (30). Thus, food insecurity could place caregivers at increased risk for obesity. We conducted sensitivity analyses stratified by food insecurity (results available from authors) but found no significant differences in obesity between caregivers and noncaregivers in either food secure or food insecure households. Among caregivers, household food insecurity and obesity may not be overlapping outcomes. However, our sample size was not large enough to further stratify by sex. The differential effects of caregiving for women, including potential relationships between food insecurity and obesity, should be explored in future larger population samples.

Our 3 outcomes differed in the level at which they were assessed. Food insecurity was measured at a household level (questions asked about the respondent, other adults in the household, or both), while hunger and obesity were measured for individual respondents. Our findings of food insecurity at the household level for caregivers echo prior findings of food insecurity in households with individuals with disabilities or chronic conditions (13,14). When caregivers and care recipients are in the same household, food insecurity may be especially likely. Our hunger analyses indicated that caregivers are also affected at an individual level. Additional research is needed to determine the extent to which other household members also experience hunger or whether caregivers go hungry to prioritize food for care recipients.

Future analyses with larger sample sizes are needed to further understand which caregiver households are at greatest risk of food insecurity. For example, some caregivers reported that the greatest difficulty caused by caregiving was financial, other caregivers reported different types of difficulties, and a substantial proportion reported no difficulty associated with caregiving. Stratification of these groups may indicate distinctions. Caregivers experiencing financial strain may be most likely to experience food insecurity and hunger; other groups may be at less risk. Similarly, caregivers providing care for a family member may differ from those providing care to nonrelatives. Identification of risk profiles would facilitate direction of interventions to households most at risk.

Our study has limitations. The data for our analyses came from a single state, which may not be representative of the United States as a whole. We are unaware of other states that have collected both caregiver and food security data in the same year. Oregon obtains food security data through state-added questions on the BRFSS, which means these data are not available on CDC’s BRFSS website. Other states may have used a similar approach and have access to food security data for caregivers. We certainly encourage other states to consider collecting and analyzing this information. With access to data from only one state, our sample size of caregivers was limited and precluded examination of potential interaction effects of income, sex, and health with caregiving in relation to food insecurity, hunger, and obesity. Larger data sets are needed to explore these issues. The BRFSS consists of cross-sectional data, which limits our ability to draw causal conclusions about the relationship between caregiving and food insecurity. Some caregivers may have been experiencing food insecurity or at increased risk for food insecurity before they began providing care. However, the association even in a cross-sectional analysis suggests that careful attention to the nutritional profile of households with family caregivers may be needed, including screening for household food insecurity.

As the US population ages, the need for informal caregiving will continue to grow (16). Caregivers play a critical role in allowing care recipients to remain in their homes and communities as long as possible rather than in more costly nursing home settings (16). Food insecurity and hunger among caregivers may limit their long-term ability to provide care, with substantial effects on well-being and health care expenditures for caregivers and care recipients alike. Therefore, addressing food insecurity and its associated risks and consequences among caregivers is a public health priority.
